# The Mediating Role of Time Perspective in the Relationship between Chronotype and Suicide in Bipolar Disorder

**DOI:** 10.3390/bs12120492

**Published:** 2022-12-02

**Authors:** Mahmut Onur Karaytuğ, Lut Tamam, Mehmet Emin Demirkol, Zeynep Namlı, Mahmut Gürbüz, Caner Yeşiloğlu, Özge Eriş Davut

**Affiliations:** 1 Department of Psychiatry, Faculty of Medicine, Çukurova University, Balcali Campus, Adana 01330, Turkey; 2 St. Elisabeth Krankenhaus Klinik Fur Psychiatrie Und, Psychotherapie, 45891 Gelsenkirchen, Nordrhein Westfalen, Germany; 3 Department of Psychiatry, Ahi Evran University, Kirsehir 40100, Turkey; 4 Department of Psychiatry, Hatay Training and Research Hospital, Antakya 31001, Turkey

**Keywords:** bipolar disorder, time perspective, chronotype, suicidality

## Abstract

(1) Background: Suicide in patients with bipolar disorder (BD) is related to the chronotype of the person from a biological perspective. However, it is not known whether there is a relationship between suicide and psychological time in BD. The aim of our study was to evaluate the relationship between time perspective (TP) and suicide and the effect of TP on the relationship between suicide and chronotype in euthymic patients with BD. (2) Methods: We included 150 BD patients and 84 healthy controls in this cross-sectional study. We administered the Young Mania Rating Scale (YMRS), Hamilton Depression Rating Scale (HDRS), Beck Scale for Suicidal Ideation (BSSI), Zimbardo Time Perspective Inventory (ZTPI), and Morning–Evening Questionnaire (MEQ). (3) Results: There was a statistically significant difference between the median scores of past negative TP, present fatalistic TP, future TP, and MEQ total score (*p* < 0.001, *p* < 0.001, *p* = 0.010, and *p* = 0.020, respectively). There was a significant correlation between past negative TP, future TP, MEQ scores, and BSSI scores in the patient group (*p* < 0.001, *p* = 0.018, and *p* = 0.028, respectively). An inverse and significant relationship between the MEQ total score and BSSI score and TP types had a mediator role in this relationship. (4) Conclusions: Our study shows that TP, which evaluates time from a psychological perspective, has a direct relationship with suicidal ideation and a mediating role in the relationship between chronotype and suicide. According to our results, we can conclude that ZTPI can also be used to evaluate the risk of suicidality in patients with BD. Appropriate therapy methods for TP may help to prevent some suicide attempts.

## 1. Introduction

Patients with bipolar disorder (BD) have a shorter life expectancy than the general population [1]. Some of the early deaths in these patients are due to unnatural causes such as suicide, accidents, and homicide [2]. The risk of suicide-related death is 10–30 times higher in patients with BD compared to the general population [3]. Risk factors for suicidal ideation in patients with BD include a family history of mood disorders, severe depression, psychotic symptoms, past suicide attempts, alcohol abuse or dependence, panic spectrum symptoms, youth age, and early onset of the disease [4].

Evidence for the relationship between human behavior and time has been increasing recently. In addition, studies show that time is an essential factor that causes behavioral differences in individuals [5,6,7]. In traditional biological approaches, chronotype is used to investigate the effect of time on behavior [8]. Chronotype is generally classified as morning type, evening type, and intermediate type. Evening-type individuals get up later and go to bed later than morning-type individuals. They show their best physical and mental performance during the day 2–3 h later than morning-type individuals [9]. An increasing number of studies on chronotype show that evening type is more likely in many psychiatric disorders, including patients diagnosed with BD [10,11,12]. Earlier at the onset of illness, higher rates of rapid cycling, at least one suicide attempt, and more past psychotic symptoms have been found in patients with evening type [13]. In addition, previous studies have shown that evening-type BD is associated with more self-harm and suicidality than other chronotypes [14,15]. However, from a psychological perspective, whether there is a relationship between time and suicide in BD is not known.

Many studies argue that time perspective is a fundamental process influenced by factors such as culture, religion, social class, education, family, and age and is the most potent factor in human thought, emotion, and behavior. Zimbardo evaluated the relationship of individuals with time from a psychological perspective and defined time perspective (TP) as an indicator of the way an individual reacts to the world in which different temporal dimensions, including past negative TP, past positive TP, present hedonistic TP, present fatalistic TP and future TP, regulate the relationships between personal and social experiences [16]. One study reported that depressive symptomatic individuals with future TP predominance had a lower suicidal tendency than non-dominant individuals [17]. It has been shown that past negative TP and present fatalistic TP scores of individuals with severe suicidal ideation and who have attempted suicide are significantly higher [18,19]. In contrast, those with lower future TP and present hedonistic TP scores have more severe suicidal ideation [20].

According to Yufit, suicide is primarily a result of the distortion of an individual’s TP [21]. MacLeod et al. presented that suicidal individuals had an impaired ability to produce positive future thoughts and that lack of positive expectations about the future distinguished suicidal and non-suicidal groups [22,23].

Studies linking chronotypes and future TP are available in the literature [8,24]. Morning type is positively associated with future TP and negatively associated with present hedonistic TP [25]. A positive attitude towards the past is associated with the morning type, and a negative attitude towards the past is associated with a tendency towards the evening type [25]. Furthermore, individuals with evening-type and present orientation are at greater risk of developing behavioral problems [16,26,27]. Previous studies have shown that past positive TP is associated with the morning type, whereas past negative TP is associated with the evening type [25]. However, our literature review found that the possible effect of the chronotype–TP relationship on suicidal ideation and behavior in BD has not been investigated.

Our study aimed to investigate the relationship between TP types and suicidal ideation in patients with BD and whether some TP types mediate the relationship between chronotype and suicide. The first of our hypotheses in this study is that the presence of past negative TP and present fatalistic TP is associated with suicide in patients with BD. Our second hypothesis is that TP types have a mediating role in the relationship between chronotype and suicide.

## 2. Method

### 2.1. Sample and Procedure

The study included 163 literate patients between the ages of 18 and 65 who were admitted to Çukurova University Faculty of Medicine Psychiatry Outpatient Clinics between 1 November 2021 and 15 June 2022, who were diagnosed with BD, and who did not have neurocognitive disorders or mental retardation. Patients with a Hamilton Depression Rating Scale (HDRS) score of 7 or less, a Young Mania Rating Scale (YMRS) score of 12 or less, and no history of any episode or hospitalization in the last three months were considered euthymic and in remission [28,29]. The first author interviewed all participants according to DSM-5 (Diagnostic and Statistical Manual of Mental Disorders) criteria [30]. We excluded nine patients who were not in the euthymic period according to the clinical interview and HDRS or YMRS scores and four patients with a diagnosis of any personality disorders from the patient group.

**Figure 1:**
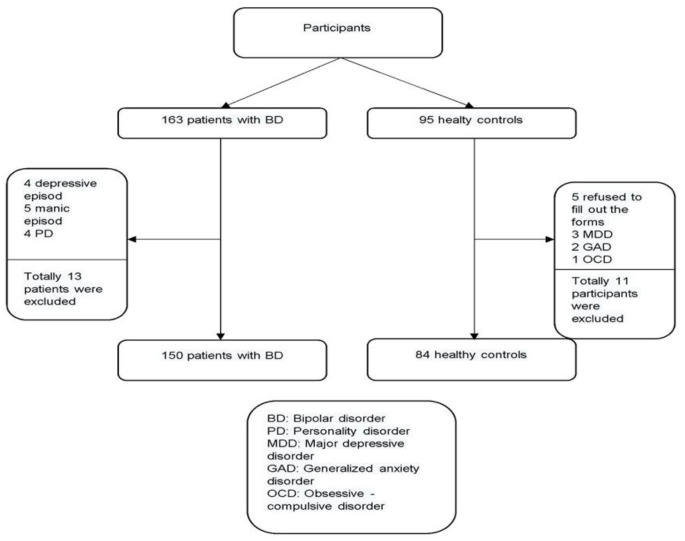
The flowchart for enrolment of the participants.

We included 95 literate hospital employees and their relatives who reported no history of psychiatric disorders and had similar sociodemographic characteristics to the patients as the control group. Five participants who refused to fill out the forms and six diagnosed with a mental disorder were excluded from the control group. The study was continued with 150 patients diagnosed with BD and 84 healthy controls. The study flow-chart is presented in Figure 1.

Each participant was allocated approximately 60 min for a clinical interview and completion of the scales. Sociodemographic data were collected with the help of a data form created by us. The interviewer explained the points that the participants did not understand.

The ethics committee approval of the study was granted by Çukurova University Faculty of Medicine, Non-Interventional Clinical Research Ethics Committee (meeting number 115, decision number 15, dated 1 October 2021). Informed written consent was obtained from all participants before the study. The study was conducted in accordance with the Declaration of Helsinki.

### 2.2. Measures

#### 2.2.1. Sociodemographic and Clinical Data Form

The data form developed by the researchers included sociodemographic data such as gender, age, education level, occupation, marital status, and information about the disease process such as duration of illness, number of hospitalizations, medication used, and suicide attempts.

#### 2.2.2. The Young Mania Rating Scale (YMRS)

It is a scale consisting of 11 items, each with 5 levels of severity, and rates the core symptoms of mania from mild to severe. Scoring is based on the patient’s reports and the clinician’s observations during the interview. However, the clinician’s opinion is prioritized [31]. In a Turkish validity and reliability study, Cronbach’s alpha value was 0.79 [32].

#### 2.2.3. The Hamilton Depression Rating Scale (HDRS)

It is a scale of 17 questions that evaluates depressive symptoms on 3 or 5 graded dimensions. High scores indicate increased severity of depressive symptoms, completed by the clinician. Scores of 7 and below indicate the absence of depression [33]. In the Turkish validity and reliability study, Cronbach’s alpha value was reported to be 0.75 [34].

#### 2.2.4. The Beck Scale for Suicidal Ideation (BSSI)

This scale evaluates the severity of suicidal ideation in five sections: characteristics of life/death attitude, characteristics of suicidal ideation/desire, characteristics of the intended attempt, a realization of the intended attempt, and background factors. The clinician completes it. High scores reflect the severity of suicidal ideation [35]. The Turkish validity and reliability study determined Cronbach’s alpha value as 0.84 [36].

#### 2.2.5. The Zimbardo Time Perspective Inventory (ZTPI)

The questionnaire developed by Zimbardo and Boyd consists of 56 questions and 5 sub-dimensions: past positive, past negative, present hedonistic, present fatalistic, and future. The scale is a 5-point Likert type. The validity and reliability study reported that the reliability coefficient varied between 0.70 and 0.80 [16]. Akırmak conducted the validity and reliability study of the scale in Turkey. In the study conducted among the Turkish sample, Cronbach’s alpha consistency coefficient was 0.74 for past positive, 0.84 for past negative, 0.78 for present hedonistic, 0.68 for present fatalistic, and 0.75 for future orientation [37].

#### 2.2.6. The Morningness–Eveningness Questionnaire (MEQ)

It consists of 19 questions that question people in terms of their lifestyle, sleep-wake patterns, and performance. It is a self-report-type evaluation scale [38]. A total score of 16–41 is classified as ‘evening type’, 42–58 as ‘intermediate type’, and 59–86 as ‘morning type’. The reliability study of the Turkish version of the questionnaire was conducted in 2005 [39].

## 3. Statistical Analysis

In summarizing the data obtained from the study, descriptive statistics were tabulated as mean ± standard deviation or median, minimum, and maximum depending on the distribution for continuous (numerical) variables. Categorical variables were summarized as numbers and percentages. The normality of the numerical variables was checked via Shapiro–Wilk, Kolmogorov–Smirnov, and Anderson–Darling tests.

In comparisons of differences between categorical variables according to groups (patient and control), the Pearson Chi-Square test was used in 2 × 2 tables with expected values of 5 and above, Fisher’s Exact Test was used in tables with expected values below 5, and the Fisher Freeman Halton test was used in RxC tables with expected values below 5.

In comparing two independent groups (patient and control), the Mann–Whitney U test was used when the numerical variables did not show normal distribution.

In comparisons of more than two independent groups (patient and control), the Kruskal–Wallis H test was used when the numerical variables were not normally distributed. In nonparametric tests, differences between groups were evaluated with the Dwass–Steel–Critchlow–Fligner test.

We used Cramer’s V coefficient to determine the effect size of the relationships between qualitative variables and the r effect size to determine between quantitative variables evaluated with the Mann–Whitney U test.

Spearman’s Rho correlation coefficient was used to examine the relationships between numerical variables when the variables did not show normal distribution.

Factors affecting the BSSI score were analyzed with univariate and multiple linear regression models. The variables included in the multiple linear regression model were selected as variables with a statistical *p*-value less than 0.250 in univariate results and clinically affecting the dependent variable. On the other hand, the variance inflation factor (VIF) value was used to check whether there was a multicollinearity problem among the independent variables. The multiple models did not include the variable “present fatalistic TP” due to the multicollinearity problem.

The mediating role of TP types in the relationship between chronotype and BSSI was examined via mediation analysis. A robust maximum likelihood (MLR) parameter estimation method was used. The endogenous variable (BSSI), the exogenous variable (chronotype), and the mediators (TP types) were continuous variables.

In the mediation analysis, the basic model, that is, whether the exogenous variable predicted the endogenous variable significantly without including the mediator variable in the analysis, was tested (basic model). Then, the model examined direct and indirect effects by adding the mediator variable. In mediation analyses, each mediator was considered separately, the mediator role of these variables was examined, and five mediation analyses were performed.

The mediator role of TP types was tested with path analysis using the MPLUS 7.4 program. Other statistical analyses were performed with Jamovi (Version 2.2.5.0) and JASP (Version 0.16.1) programs, and the significance level was taken as 0.05 (*p*-value).

## 4. Results

Table 1 presents the sociodemographic data of the sample. There was a statistically significant difference between the patient and control groups regarding employment status (*p* = 0.006); the proportion of non-workers in the patient group was significantly higher than in the control group. There was no statistically significant difference between the groups regarding age, gender, education level, marital status, place of residence, smoking and alcohol use, and social support.

Table 2 details the clinical characteristics of the patient and control groups. The median number of depressive episodes was 2.0 [0.0–10.0], and the median number of manic episodes was 1.0 [1.0–6.0]. Manic and depressive episodes were predominant in 40% and 60% of the patients. The median number of hospitalizations was 2.0 [0.0–8.0]. The median duration of disorder was 8.0 [1.0–30.0] years (Table 2).

The difference between the percentages of history of physical illness and the presence of mental disorders in the family were statistically significant (Table 2; *p* = 0.048 and *p* < 0.001, respectively). The rates of history of physical illness and family history of mental disorder were significantly higher in the patient group than in the control group.

Table 3 presents that there was a statistically significant difference between the median scores of past negative TP, present fatalistic TP, and future TP sub-dimensions of the ZTPI (*p* < 0.001, *p* < 0.001, and *p* = 0.010, respectively). While the median scores of the past negative TP and future TP sub-dimensions were significantly higher in the patient group compared to the control group, the median score of the present fatalistic TP sub-dimension was higher in the control group (Table 3).

There was a statistically significant difference between the MEQ total score medians and MEQ classification rates according to the groups (*p* = 0.020 and *p* < 0.001, respectively). The median MEQ total score of the individuals in the patient group was significantly higher than the control group. Similarly, the proportion of evening-type and morning-type people in the patient group was significantly higher than in the control group. In comparison, the proportion of intermediate type was higher in the control group than in the patient group (Table 3).

When the factors affecting the BSSI of the individuals in the patient group were examined in Table 4, all variables included in the model influenced suicide in univariate results (*p* < 0.05 for each). In contrast, past negative TP, future TP, and MEQ total scores were found to be effective in the multiple models (*p* < 0.001, *p* = 0.018, and *p* = 0.028, respectively) (Table 4).

Table 5 demonstrates that there was a statistically significant, linear, and same-directional relationship between the BSSI of the patient group and the past negative TP and present fatalistic TP sub-dimension scores (r = 0. 540 *p* < 0.001 and r = 0.433 *p* = <0.001, respectively), and there was an inverse relationship between the past positive TP, present hedonistic TP, and future TP subscale scores (Table 5; r = −0.360 *p* < 0.001, r= −0.337 *p* < 0.001 and *p*= −0.371 *p* < 0.001, respectively). A statistically significant, linear, and an inverse relationship was observed between the BSSI and MEQ total score in the patient group (Table 5; r = −0.228 *p* = 0.005).

## 5. Mediation Analysis

### Sub-Problem

What is the mediating role of TP types in the relationship between chronotype and suicidal ideation in patients with BD?

Within the scope of this sub-problem, the mediating role of TP types in the relationship between chronotype and BSSI in patients with BD was addressed, and a total of five mediation analyses were applied (Model 1–Model 5). In this context, we first examined the direct effect of chronotype on BSSI without adding mediator variables (TP types) to the model. In this basic model, it was observed that chronotype significantly predicted BSSI in a negative direction (β = −0.287; *p* = 0.002; R^2^ = 0.08).

**Figure 2:**
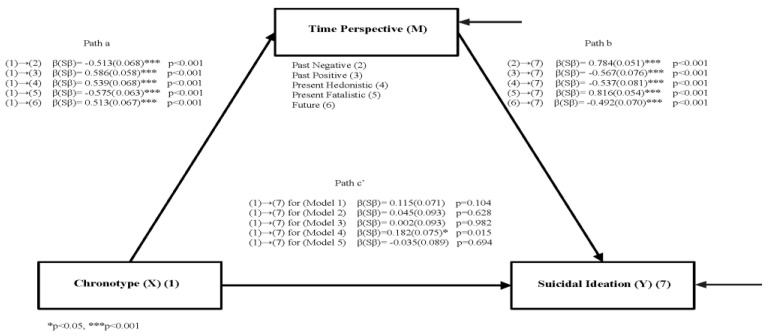
The mediating role of time perspective on the relationship between chronotype scores and suicidal ideation.

The path diagram, including the standardized path coefficients and standard errors of the mediation analyses in which TP types were added, is presented in Figure 2. In all models in Figure 2, the effect of chronotype on the mediator (path a) and mediator on BSSI (path b) was significant. The effect of chronotype on BSSI (path c’) was significant only in Model 4 (*p* = 0.015); it was not significant in the other models (*p* > 0.05 for each model). In addition, chronotype negatively predicted the mediator in Model 1 and Model 4, and the mediator negatively predicted BSSI in Model 2, Model 3, and Model 5.

In addition, the direct and indirect effects obtained from these models are presented in Table 6. The indirect effects obtained in all models were significant (*p* < 0.001). The standardized path coefficients (β) for the mediator role of past negative TP, past positive TP, present hedonistic TP, present fatalistic TP, and future TP, respectively, were as follows: −0.402, −0.332, −0.289, −0.289, −0.469, and −0.252. The explained variance (R2) obtained in all models are as follows for past negative TP, past positive TP, present hedonistic TP, present fatalistic TP and future TP respectively: 26%, 34%, 29%, 33%, and 26% for mediator; 54%, 29%, 29%, 29%, 53%, and 26% for BSSI.

In summary, past negative TP (Model 1), past positive TP (Model 2), present hedonistic TP (Model 3), and future TP (Model 5) were found to play a full mediator role in the relationship between chronotype and BSSI, because the effect of chronotype on the mediator (path a) and mediator on BSSI (path b) was found to be significant, but the effect of chronotype on BSSI (path c’) was not significant. However, present fatalistic TP (Model 4) was found to partially mediate the relationship between chronotype and BSSI because the effects of chronotype on present fatalism (path a), present fatalism on BSSI (path b), and chronotype on BSSI (path c’) were found to be significant.

## 6. Discussion

The most important finding of this study is the demonstration of the mediating role of TP in the relationship between chronotype and suicide in patients with BD. Our study found that past negative TP, past positive TP, present hedonistic TP, and future TP had a fully mediating role, and present fatalistic TP partially mediated the relationship between chronotype and suicide. These results suggest that interventions aimed at time perspectives may reduce the risk of suicide in patients with BD.

Our study reveals the full mediating role of past negative TP in the same direction and future TP in the opposite direction in the relationship between chronotype and suicide in euthymic patients with BD. The study conducted by Laghi et al. showed that past negative TP was associated with suicidal ideation [18]. Yufit stated that suicide is primarily a result of unhealthy TPs. According to Yufit, suicide is associated with a negative preoccupation with the past and a reduction of the future or detachment from the future [40]. According to Yufit, fixation on the past impoverishes emotional resources and reduces one’s investment in the future. In addition, it prevents the person from developing problem-solving skills, which leads to a loss of hope for the future. This reinforces feelings of helplessness and being trapped in one’s painful situation [41]. This process may lead to negative views of oneself, such as dissatisfaction, guilt and shame, and suicidal tendencies. In addition, our study determined the opposite full mediating role of past positive TP in the relationship between chronotype and suicide. According to Holman et al., people with higher past positive TP scores than ZTPI remember positive memories more and experience behavioral problems less [42]. Van Beek et al. reported that past positive TP might be protective against psychopathology [43]. In addition, some researchers reported that those who scored high on this dimension tended to recall rejection and adverse events less and that past positive TP showed a positive relationship with life satisfaction level [44,45]. These results, which are compatible with our study, suggest that past positive TP, that is, gaining a positive perspective towards the past, will reduce the risk of suicide. Another finding of our study is that present hedonistic TP decreases suicidal ideation in the relationship between chronotype and suicide with a full mediator role in the opposite direction. According to Zimbardo and Boyd, present hedonistic TP is mainly associated with mindfulness, extroversion, openness, and well-being [16]. These characteristics may explain the decrease in suicidal ideation associated with present hedonistic TP.

It has been shown in various studies that ZTPI subscales and chronotypes may be associated with suicide [13,17]. However, to our knowledge, this relationship has been examined for the first time in patients diagnosed with BD. Our study determined that suicidal ideation increased as past negative TP and present fatalistic TP scale scores increased and as future TP, past positive TP, and present hedonistic TP scale scores decreased. In addition, suicidal ideation increased as the MEQ scale score decreased, as a tendency towards evening type occurred. These results support our hypothesis in the relationship between ZTPI, MEQ, and BSSI. When we examined the relationship between these parameters with the regression model, it was observed that MEQ, past negative TP, and future TP were more effective on suicidal ideation. These results indicate that patients with BD who wake up later, sleep later, and focus more on the negative aspects of past experiences are more likely to have suicidal ideation. It also shows that patients with higher future TP scores, that is, those who focus more on the possible consequences of current behaviors, have a decrease in suicidal ideation.

According to Zimbardo, past negative TP and present fatalistic TP are associated with several psychopathologies [16]. Our literature review found few studies on the relationship between BD and TP. Oyanadel and Buela-Casal revealed that the tendency to past negative TP was higher in patients with BD than in the healthy population. Oyanadel and Buela-Casal have also found a higher tendency to past positive TP in the healthy group, and no differences were found between the healthy population and patients with BD in other subscales [46]. In our study, past negative TP was higher with a moderate effect size, future TP with a small effect size in patients with BD, and present fatalistic TP with a moderate effect size in healthy individuals. The higher present fatalistic TP in healthy individuals may be related to the fact that TP is influenced by culture and religion [47]. The rates of past negative TP, past positive TP, and future TP in patients with BD may be related to the course of the disease, the type of the last episode, and the number of episodes. In the present study, we evaluated euthymic patients but did not make additional analyses according to episode types. Thus, more studies are needed to confirm our results.

There are some conflicting results on the relationship between chronotype and BD. According to some studies, patients with BD are more likely to be evening type [10,11,48]. However, in some studies, BD was not found to be specifically associated with any chronotype [12,49]. In our study, MEQ total scores differed with small effect size, and chronotype types differed with medium effect size between BD patients and healthy controls. Our results could not determine whether patients with BD were more prone to eveningness or morningness. More studies with large samples are needed to clarify this relationship.

In our study, the difference in the frequency of unemployment, physical illness, and family history of mental disorders between patients with BD and healthy controls can be ignored due to its small effect size.

It may be possible to change time orientation in severe mental disorders. One study reported that group therapy to support future orientation reduced suicidal ideation [50,51]. In another pilot study, positive results were obtained in future-oriented group therapy with a positive outlook for patients with major depressive disorder [50]. In addition, TP therapy proposed by Zimbardo can also be used in patients with suicidal BD. In this therapy, the strict TP is first identified. Then, the person is told about the importance of time and the goal of moving from the dominant TP to a balanced state. The aim is to reconstruct a more caring past, to experience a more satisfying present, and to imagine a future that will not cause anxiety [52].

## 7. Limitations and Future Directions

The study’s results should be considered in light of some limitations. Since the present study was cross-sectional, a causal relationship cannot be established regarding the results. The selection of the control group from a narrow sample may influence the study results as it may not represent the general healthy population. The absence of a ‘negative future’ subscale in the ZTPI did not allow an evaluation of this factor. This is the first study on TP and suicidality in patients with BD. Future research should extend the current findings by adding other variables such as different cultures, religious beliefs, manic and depressive episodes, and episode severity, which may mediate the effects of TP on suicide. In addition, there is a need for longitudinal studies that can give an idea about whether the results we obtained vary according to the treatment and whether there is continuity.

## 8. Conclusions

Patients with BD are a high-risk group for suicide, and it is essential to predict and prevent suicide in these patients. Our study revealed the relationship between chronotype, TP, and suicide in patients with BD. In this study, especially evening-type chronotype, past negative TP, and future TP were found to be associated with suicide. We also found that TPs mediate between chronotype and suicidal ideation. In light of these findings in our study, it will be possible to develop personalized treatment plans by identifying patients with a high risk of suicide with the ZTPI. This scale can be easily applied in outpatient clinic conditions.

## Figures and Tables

**Table 1:** Sociodemographic features of the participants.

	Patients (n = 150)	Control (n = 84)	*p*
Age ^†^	42.3 ± 12.1	43.2 ± 11.5	
^§^	43.0 [22.0–65.0]	41.0 [22.0–67.0]	0.841
Gender ^‡^			
Male	71 (47.3)	47 (56.0)	0.259
Female	79 (52.7)	37 (44.0)	
Education ^‡^			
Primary School	36 (24.0)	26 (31.0)	0.276
Secondary School	77 (51.3)	44 (52.4)	
University	37 (24.7)	14 (16.7)	
Marital Status ^‡^			
Single	86 (57.3)	40 (47.6)	0.196
Married	64 (42.7)	44 (52.4)	
Employment Status ^‡^			
Employed	43 (28.7)	40 (47.6)	0.006
Unemployed	107 (71.3)	44 (52.4)	Cramer’s V = 0.190
Residence ^‡^			
Urban	77 (51.3)	46 (54.8)	0.713
Rural	73 (48.7)	38 (45.2)	
Smoking, presence ^‡^	72 (48.0)	31 (36.9)	0.133
Alcohol use, presence ^‡^	42 (28.0)	25 (29.8)	0.892
Social Support, presence ^‡^	148 (98.7)	83 (98.8)	0.999

^‡^: n (%), Pearson Chi-Square, Fisher’s Exact or Fisher Freeman Halton test was used. ^†^: mean ± standard deviation, ^§^: median [min–max], Mann–Whitney U test was used.

**Table 2:** Clinical characteristics of the patients and controls.

	Patients (n = 150)	Control (n = 84)	*p*
Number of depressive episodes ^§^	2.0 [0.0–10.0]	-	-
Number of manic episodes ^§^	1.0 [1.0–6.0]	-	-
Predominant polarity ^‡^			
Manic	60 (40.0)	-	-
Depressive	90 (60.0)	-	
Number of hospitalizations ^§^	2.0 [0.0–8.0]	-	-
Substance use, presence ^‡^	0 (0)	-	-
History of physical illness, presence ^‡^	60 (40.0)	22 (26.2)	0.048 Cramer’s V = 0.139
Family history of psychiatric disorder, presence ^‡^	85 (56.7)	23 (27.4)	<0.001 Cramer’s V = 0.282
Duration of disorder (year) ^§^	8.0 [1.0–30.0]	-	-

^‡^: n (%), Pearson Chi-Square test was used. ^§^: median [min–max].

**Table 3:** The scale scores of the participants.

	Patients (n = 150)	Control (n = 84)	Effect Size *	*p*
Beck Scale for Suicidal Ideation ^§^	1.0 [0.0–15.0]	-		-
Zimbardo Time Perspective Inventory ^§^				
Past negative	17.5 [13.0–43.0]	16.0 [13.0–19.0]	0.428	<0.001
Past positive	28.0 [15.0–37.0]	27.0 [17.0–34.0]		0.204
Present fatalistic	18.0 [11.0–43.0]	25.0 [15.0–42.0]	0.364	<0.001
Present hedonistic	41.0 [19.0–54.0]	43.0 [26.0–55.0]		0.324
Future	40.5 [27.0–51.0]	39.5 [28.0–48.0]	0.169	0.010
MEQ total score ^§^	56.0 [19.0–85.0]	52.0 [19.0–73.0]	0.152	0.020
MEQ classification ^‡^				
Eveningness	27 (18.0)	6 (7.1)	0.304	<0.001
İntermediate	58 (38.7)	59 (70.2)		
Morningness	65 (43.3)	19 (22.6)		

^‡^: n (%), Pearson Chi-Square/Fisher Freeman Halton test was used. ^§^: median [min–max], Mann–Whitney U test was used. *: r effect size. MEQ: Morningness–Eveningness Questionnaire.

**Table 4:** The regression models for variables related to Beck Scale for Suicidal Ideation.

	Beta [%95 CI]	Adj. Beta [%95 CI]	*p*-Value
Past negative TP	0.362 [0.314–0.411]	0.803 [0.696–0.911]	<0.001
Past positive TP	0.031 [−0.081–0.143]	0.040 [−0.104–0.183]	0.587
Present hedonistic TP	−0.005 [−0.070–0.061]	−0.010 [−0.150–0.130]	0.892
Future TP	−0.068 [−0.153–0.017]	−0.093 [−0.210–0.024]	0.017
MEQ total score	0.043 [0.012–0.075]	0.139 [0.037–0.240]	0.008
Age	−0.002 [−0.029–0.025]	−0.005 [−0.090–0.079]	0.901
Male	0.287 [−0.356–0.930]	0.038 [−0.047–0.123]	0.380
Primary School	0.126 [−0.815–1.067]	0.015 [−0.095–0.124]	0.792
Secondary School	0.029 [−0.787–0.845]	0.004 [−0.104–0.111]	0.944
Single	−0.206 [−0.860–0.447]	−0.027 [−0.113–0.059]	0.535

MEQ: Morningness–Eveningness Questionnaire. TP: time perspective.

**Table 5:** Correlations among ZTPI, MEQ, and BSSI in the patient group.

	Beck Scale for Suicidal Ideation (BSSI)	
	r	*p*
Zimbardo Time Perspective Inventory (ZTPI)		
Past negative	0.540	<0.001
Past positive	−0.360	<0.001
Present fatalistic	0.433	<0.001
Present hedonistic	−0.337	<0.001
Future	−0.371	<0.001
MEQ total score	−0.228	0.005

Spearman’s rho correlation coefficient was used. MEQ: Morningness–Eveningness Questionnaire.

**Table 6:** The mediating role of time perspective on the relationship between chronotype scores and suicidal ideation.

Model		Pathway	Std. Path Coefficient (β)	Std Error (S_β_)	*p*-Value
Basic model (without ZTPI)		Chronotype → BSSI	−0.287	0.091	0.002
Model I. Mediation Analysis (Past negative mediator)	Direct effect	Chronotype → BSSI	0.115	0.071	0.104
	Indirect effect	Chronotype → Past negative → BSSI	−0.402	0.065	<0.001
Model II. Mediation Analysis (Past positive mediator)	Direct effect	Chronotype → BSSI	0.045	0.093	0.628
	Indirect effect	Chronotype → Past positive → BSSI	−0.332	0.063	<0.001
Model III. Mediation Analysis (Present hedonistic mediator)	Direct effect	Chronotype → BSSI	0.002	0.093	0.982
	Indirect effect	Chronotype → Present hedonistic → BSSI	−0.289	0.063	<0.001
Model IV. Mediation Analysis (Present fatalistic mediator)	Direct effect	Chronotype → BSSI	0.182	0.075	0.015
	Indirect effect	Chronotype → Present fatalistic → BSSI	−0.469	0.070	<0.001
Model V. Mediation Analysis (Future mediator)	Direct effect	Chronotype → BSSI	−0.035	0.089	0.694
	Indirect effect	Chronotype → Future → BSSI	−0.252	0.052	<0.001

ZTPI: Zimbardo Time Perspective Inventory BSSI: Beck Scale for Suicidal Ideation.

## Data Availability

The data presented in this study are available on request from the corresponding author.

## Glossary

BD	Bipolar disorder
TP	Time perspective
HDRS	Hamilton Depression Rating Scale
YMRS	Young Mania Rating Scale
ZTPI	Zimbardo Time Perspective Inventory
MEQ	Morning–Evening Questionnaire
BSSI	Beck Scale for Suicidal Ideation
DSM-5	Diagnostic and Statistical Manual of Mental Disorders
